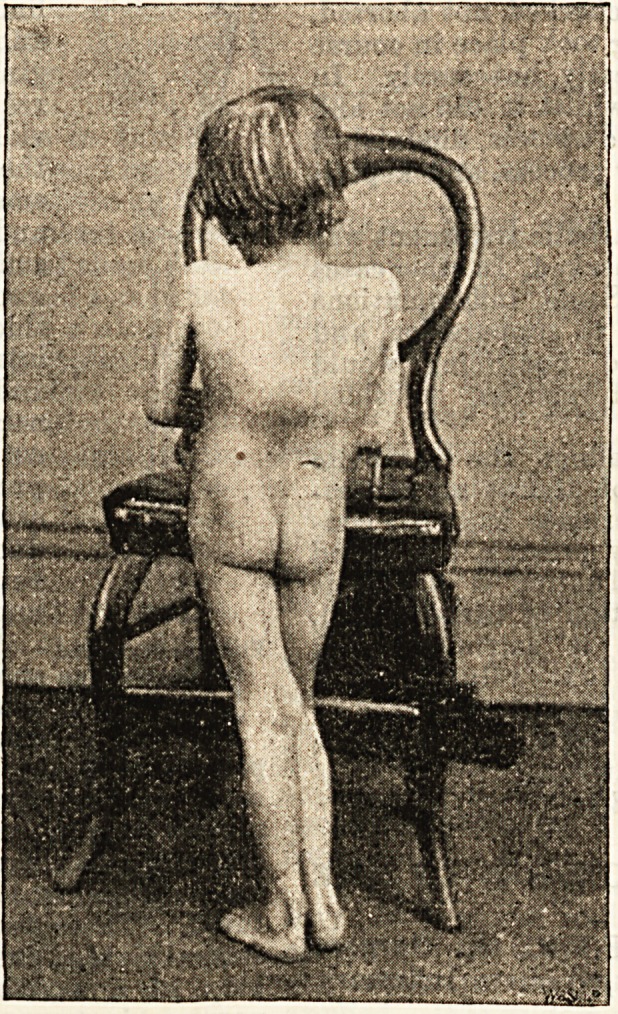# Some Simple Uses of Carbolic Acid. I

**Published:** 1893-07-01

**Authors:** A. G. Miller

**Affiliations:** Lecturer on Clinical Surgery, and Surgeon to the Edinburgh Royal Infirmary


### THE HOSPITAL CLINIC.

[*The Editor will be glad to receive offers of co-operation and contributions from members of the profession. All letters should be addressed to* THE EDITOR, THE LODGE, PORCHESTER SQUARE, LONDON, W.]

### SOME SIMPLE USES OF CARBOLIC ACID.—I.

By A. G. MILLER, M.D., F.R.C.S.E., Lecturer on Clinical Surgery, and Surgeon to the Edinburgh Royal Infirmary.

In 1865 I had the good fortune to be house surgeon to one of the most advanced surgeons of his day—the late Professor James Spence. He gave great care and attention to the dressing of his operation cases and compound fractures.

Now every surgeon knows that compound fractures are test cases. They are very difficult to treat properly. They test the skill in antiseptics of the modern surgeon; they tested the skill of the surgeon still more before the introduction of antiseptics.

Mr. Spence always had a number of compound fractures "on hand." They were mostly of the leg in my time I think. These were laid in state on bent McIntyre splints. The splints were covered with mackintosh or gutta-percha tissue, which was changed daily. The wounds which had previously been slit up as freely as possible, and all fragments of bone and all bruised and dirty tissue removed, were syringed once or twice daily with a solution of permanganate of potash, and lightly covered with lint that had been dipped in a weak solution of sodium chlorate. The result of this treatment was very good. Many legs were saved from amputation, and there was very little pyæmia in our wards.

In 1867 I went to Glasgow to see what was being done with antiseptics. I had read Sir Joseph Lister's writings on the subject, and wanted to see for myself what was being done. What I saw astonished me very much, and confirmed what I had read of the wonders wrought by carbolic acid. Under a covering of carbolic oil or carbolic putty were open compound fractures without a drop of pus about them. Bare bone was demonstrated to be alive by the pink spots of vascular life that first shone through, and then gave birth to granulations. Above all, what pleased me most was that the patients were cheery and happy, pyæmia seemed to be a thing unknown, and amputation was threatening to become a lost art.

This reminds me of another experience I had last summer. I was in Glasgow again, visiting a distinguished surgeon. In Professor Macewen's wards I saw a man who had sustained a compound comminuted fracture of both bones of the forearm. I was told that the injury (a machine accident) had been inflicted a fortnight before; that the skin had been thoroughly slit up, and all fragments of bone and shreds of dirty or bruised muscle removed; that the bones had been stitched, the muscles and tendons united, and the skin brought together as thoroughly as possible. The dressing put on at the close of the operation, which had lasted fully an hour, was untouched and perfectly dry and clean, and no antiseptic but carbolic acid had touched the wound. The patient could move his fingers freely, and had no pain or discomfort. I heard from Professor Macewen afterwards that the dressing was not removed till about five weeks after the operation, when there was a mere superficial wound visible. The man has an excellent serviceable arm. This I consider one of the grandest successes of antiseptics. Those who have had experience of dirty, battered machinery and railway accident cases can appreciate the triumph of such a case as this.

When I returned to Edinburgh in 1867 it was with a devotion to Listerism and carbolic acid that has never flagged, but always grown, as knowledge has been expanded by experience. I began at once with the carbolic lotion, carbolic oil, and carbolic putty to work wonders, at least in my own estimation. I remember my first case well. It was in 1867 that a boy about nine, with Pott's curvature of the spine and lumbar abscess, was brought to me at the dispensary by his mother, who was in great distress because she had taken him to the infirmary, and my old master, Mr. Spence, had advised that nothing should be done to him, because opening the abscess would only hasten his death. And I believe he was quite right. When I saw this case, having learned Mr. Lister's treatment of suppuration, I at once undertook to treat it with far greater confidence than I would now were I to employ the same means. After washing the part well with carbolic lotion, I hung a piece of lint soaked in carbolic oil over the abscess, and lifting up the lower margin, made a small puncture with a fine bistoury. I then let the curtain fall, and the thin pus trickled away under its protection. When the discharge of pus ceased, the puncture and the skin for some distance round were covered with a thick layer of the carbolic putty laid on a thin sheet of lead. The putty dressing was changed daily under the protection of a carbolic screen for some weeks, then only every few days, at longer intervals, until the wound healed. This occurred in a few months. The back in the meantime was unsupported in any way. The child lay in bed a good deal, but was taken out in a little cart daily when possible. The little fellow recovered completely, and I have a photograph of him which shows the marked curvature and the depressed cicatrix where the abscess was opened.

The uses to which carbolic acid is put in surgery are many, such as a lotion for wounds, hands, instruments, sponges, &c., but those to which I wish to refer in this paper are perhaps not so well known. My attention was specially drawn to the matter in 1884, when Dr. Sturrock, now of Broughty Ferry, was my house surgeon.

The routine practice in my wards in all cases that required operation was to render the skin at the part to be operated on aseptic by the application of a towel soaked in carbolic lotion (one to 20), and covered with mackintosh to prevent evaporation. This practice of applying the "carbolic towel," as it was called, was carried out in septic as well as aseptic cases.

I had noticed that the "carbolic towel," when applied for even a single night, diminished the signs of inflammation if these were present. Skin became less red, swelling went down, and tension diminished (as shown often by wrinkling of the skin), heat and pain also subsided.

We had used the "carbolic towel" for years, and had noticed this result sometimes after a single night's application; but Dr. Sturrock first markedly drew my attention to the matter, and asked my leave to experiment. We treated some cases of abscess and of septic inflammation after wounds with the "carbolic towel" only. Inflammation went down quickly and satisfactorily, and abscesses were influenced in a remarkable manner. We found that the abscess at first seemed inclined to disappear. Time, however, demonstrated that the suppurative process was only checked, not aborted, and the classic process of pointing took place in course of time, more slowly and painlessly, but surely. In other words, the carbolic lotion, whilst it did not and could not cause absorption of the abscess, diminished the activity of the inflammatory process, and kept it at a minimum.

In some wounds in which the carbolic treatment was employed formation of pus was checked. In other cases it was prevented altogether. Some inflammations that were evidently hastening on to suppuration, and in which ordinary old-fashioned poulticing would infallibly have caused pus formation, subsided completely.

This experience led me to adopt the "carbolic towel" as my routine treatment of almost all inflammations at all stages, and experience has only confirmed me in this practice by proving that good generally results. This beneficial action is due, as is now well known, to a threefold action of carbolic acid, for besides being antiseptic it is astringent and anæsthetic. Furthermore, to add to the valuable qualities of this wonderful substance, it is easily absorbed by the skin, and, moreover, is readily separated from the the water in which it is held in solution.

*(To be continued.)*

## Figures and Tables

**Figure f1:**